# Supplementary material for: Distribution and Phylogeny of Erythrocytic Necrosis Virus (ENV) in Salmon Suggests Marine Origin
Source: Viruses. 2019 Apr 18;11(4):358. doi: 10.3390/v11040358 (PMC6520742; doi:10.3390/v11040358)

Summary of results from ENV sequence enrichment

50-by or larger sequence alignments to putative ENV protein-encoding sequences with 99% or higher nucleotide identity from samples enriched for these sequences are summarized below. Green cells indicate detection of a putative ENV sequence in each of the fish samples enriched for ENV sequences, while grey cells indicate that a sequence was not found (left). The number of sequences found in individual fish from enrichments and between samples is shown in the Venn diagram (right)

SEQ ID	Herring	Chinook	Atlantic
SEQ_103	green	grey	grey
SEQ_106	green	grey	grey
SEQ_19	green	green	green
SEQ_20	grey	green	grey
SEQ_24	green	grey	grey
SEQ_30	green	grey	grey
SEQ_32	green	green	green
SEQ_4	green	grey	grey
SEQ_40	green	green	green
SEQ_46	green	green	green
SEQ_49	grey	green	grey
SEQ_5	grey	grey	green
SEQ_50	green	grey	green
SEQ_52	green	grey	green
SEQ_54	green	green	green
SEQ_57	green	grey	green
SEQ_62	green	grey	green
SEQ_63	green	grey	green
SEQ_65	grey	green	grey
SEQ_69	green	green	green
SEQ_70	green	grey	green
SEQ_73	green	grey	green
SEQ_74	green	green	green
SEQ_75	green	grey	green
SEQ_77	green	green	green
SEQ_78	green	green	green
SEQ_79	green	green	green
SEQ_80	green	green	green
SEQ_81	green	grey	grey
SEQ_86	green	green	green
SEQ_87	green	green	green
SEQ_89	green	grey	grey
SEQ_92	green	green	green
SEQ_93	grey	grey	green
SEQ_98	grey	grey	green

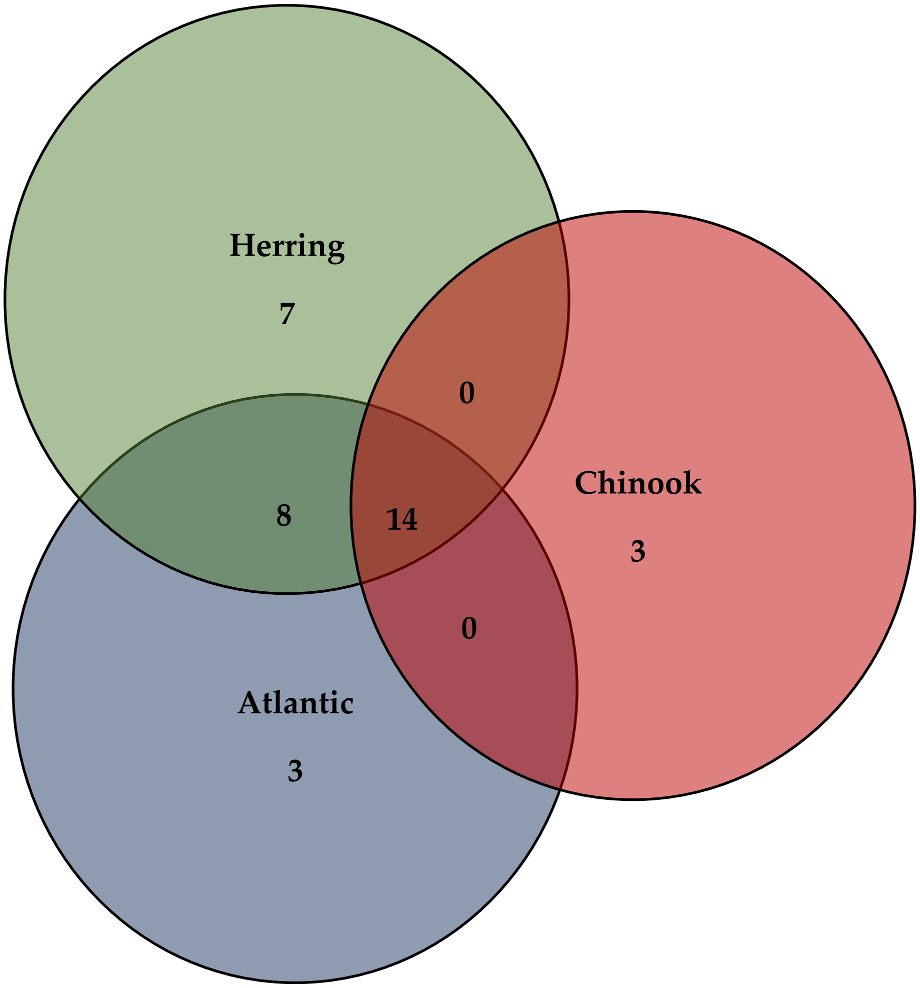

Supplement: Supplementary file 1 [file viruses-11-00358-s001.zip › viruses-475811-suppl-final/Enrichment Summary (File S4).pdf]
