# Supplementary material for: Distribution and Phylogeny of Erythrocytic Necrosis Virus (ENV) in Salmon Suggests Marine Origin
Source: Viruses. 2019 Apr 18;11(4):358. doi: 10.3390/v11040358 (PMC6520742; doi:10.3390/v11040358)
Supplement: Supplementary file 1 [file viruses-11-00358-s001.zip › viruses-475811-suppl-final/NCVOG Summary (FILE S2).docx]

**Reconstructed core gene set (47 NCVOGs) of the common ancestor of the NCLDV**

*Obtained from Yutin et al., 2009

Genes highlighted in green indicate sequences with multiple blast hits to putative ENV sequences

In total, **21/47 (43%) of NCVOG genes BLAST hit to putative ENV sequences**

| **NCVOG** | **functional category** | **NCVOG annotation** |
| --- | --- | --- |
| NCVOG0076  SEQ_92  SEQ_87 | DNA replication, recombination and repair | DNA or RNA helicases of superfamily II (COG1061) |
| NCVOG0023  SEQ_80  SEQ_40  SEQ_46  SEQ_52  SEQ_54  SEQ_57  SEQ_74 | DNA replication, recombination and repair | D5-like helicase-primase |
| NCVOG0038  SEQ_79  SEQ_50  SEQ_73 | DNA replication, recombination and repair | DNA polymerase elongation subunit family B |
| NCVOG0037 | DNA replication, recombination and repair | DNA topoisomerase II |
| NCVOG0276  SEQ_35  SEQ_76 | Nucleotide metabolism | Ribonucleotide reductase small subunit |
| NCVOG1353  SEQ_68 | Nucleotide metabolism | ribonucleoside diphosphate reductase, alpha subunit |
| NCVOG0052  SEQ_116  SEQ_47 | Virion structure and morphogenesis | disulfide (thiol) oxidoreductase; Erv1 / Alr family (pfam04777) |
| NCVOG0236 | Transcription and RNA processing | Nudix hydrolase (D10 ortholog) |
| NCVOG0262  SEQ_94  SEQ_51  SEQ_59 | Transcription and RNA processing | pfam04947, Poxvirus Late Transcription Factor VLTF3 like |
| NCVOG1164 | Transcription and RNA processing | A1L transcription factor/late transcription factor VLTF-2; pfam03295: Pox_TAA1; Poxvirus transactivator protein A1 C-terminal |

| NCVOG0271  SEQ_78  SEQ_97  SEQ_93  SEQ_70 | Transcription and RNA processing | DNA-directed RNA polymerase subunit beta |
| --- | --- | --- |
| NCVOG0274  SEQ_77  SEQ_12  SEQ_63  SEQ_69 | Transcription and RNA processing | DNA-directed RNA polymerase subunit alpha |
| NCVOG0272  SEQ_89 | Transcription and RNA processing | Transcription factor S-II (TFIIS)-domain-containing protein |
| NCVOG1117 | Transcription and RNA processing | mRNA capping enzyme large subunit |
| NCVOG1361  SEQ_53 | Uncharacterized | pfam10544, T5orf172 domain |
| NCVOG0022  SEQ_86  SEQ_75 | Virion structure and morphogenesis | NCLDV major capsid protein (pfam03340 for Poxviridae; pfam04451 for others) |
| NCVOG0249  SEQ_7  SEQ_108  SEQ_113  SEQ_41  SEQ_45 | Virion structure and morphogenesis | A32-like packaging ATPase |
| NCVOG0278 | DNA replication, recombination and repair | RuvC, Holliday junction resolvase (HJRs); cl00243. Extended Pox_A22, Poxvirus A22 family (pfam04848). |
| NCVOG1060  SEQ_91  SEQ_110  SEQ_25  SEQ_37 | DNA replication, recombination and repair | FLAP-like endonuclease XPG |
| NCVOG0319 | Nucleotide metabolism | Thymidine kinase |
| NCVOG0330 | Signal transduction regulation | RING-finger-containing E3 ubiquitin ligase (COG5432: RAD18) |
| NCVOG0261  SEQ_82  SEQ_32 | Transcription and RNA processing | Poxvirus early transcription factor (VETF), large subunit (pfam04441) |
| NCVOG0273  SEQ_44 | Transcription and RNA processing | divergent DNA-directed RNA polymerase subunit 5 |
| NCVOG0034 | DNA replication, recombination and repair | ATP-dependent DNA ligase (pfam01068, PRK01109)^a^ |
| NCVOG0004  SEQ_98 | DNA replication, recombination and repair | AP (apurinic) endonuclease family 2 – bacterial |
| NCVOG1192 | DNA replication, recombination and repair | YqaJ viral recombinase family (pfam09588) |

| NCVOG1068 | Nucleotide metabolism | dUTPase (cl00493) |
| --- | --- | --- |
| NCVOG0320  SEQ_103  SEQ_24 | Nucleotide metabolism | pfam02223: Thymidylate kinase |
| NCVOG0040 | Other metabolic functions | cd00127, DSPc, Dual specificity phosphatases (DSP); Ser/Thr and Tyr protein phosphatases |
| NCVOG1127 | Transcription and RNA processing | transcription initiation factor IIB |
| NCVOG0010  SEQ_96 | Uncharacterized | pfam02498: Bro-N; BRO family, N-terminal domain: This family includes the N-terminus of baculovirus BRO and ALI motif proteins. |
| NCVOG0211 | Virion structure and morphogenesis | myristylated IMV envelope protein (pfam02442: Lipid membrane protein of large eukaryotic DNA viruses) |
| NCVOG0035 | DNA replication, recombination and repair | NAD+ dependent DNA ligase (smart00532)^a^ |
| NCVOG0024 | DNA replication, recombination and repair | Superfamily II helicase related to herpesvirus  replicative helicase (origin-binding protein UL9), pfam03121 |
| NCVOG0036 | DNA replication, recombination and repair | DNA topoisomerase I |
| NCVOG0267 | DNA replication, recombination and repair | RNA-helicase DExH-NPH-II |
| NCVOG0009 | Host-virus interactions | pfam00653: BIR (Baculovirus Inhibitor of apoptosis protein Repeat) domain |
| NCVOG0012 | Host-virus interactions | C-type lectin: smart00034, cd03594,cd03593, pfam00059, cd00037, pfam05966 |
| NCVOG1360 | Miscellaneous | KilA domain (pfam04383); always is present at Nterminal except for mimiviruses. Sometimes is followed by a RING-finger domain |
| NCVOG1115 | Other metabolic functions | uracil-DNA glycosylase |
| NCVOG0246 | Other metabolic functions | pfam02902, Ulp1 protease family, C-terminal catalytic domain |
| NCVOG1088  SEQ_81  SEQ_21 | Transcription and RNA processing | RNA ligase (conserved in irido-, asfa- asco- and Marseille viruses) |
| NCVOG1424 | Uncharacterized | uncharacterized domain; found downstream KilA, BRO, and MSV199 domains. Also is found in some baculoviruses (gi 165969059, 18138388) |
| NCVOG1122  SEQ_95 | Virion structure and morphogenesis | Myristylated protein; pfam03003, DUF230 |
| NCVOG0256 | Other metabolic functions | IMV envelope protein p35 |
| NCVOG0329 | Other metabolic functions | UBCc, Ubiquitin-conjugating enzyme E2 (cd00195) |
| NCVOG0059 | Other metabolic functions | FtsJ-like methyltransferase family proteins (pfam01728) |

^a^Phylogenetic analysis of the DNA ligases indicates that the NAD-dependent ligase but not the ATP-dependent ligase is an ancestral NCLDV gene [1].
