# Supplementary material for: Distribution and Phylogeny of Erythrocytic Necrosis Virus (ENV) in Salmon Suggests Marine Origin
Source: Viruses. 2019 Apr 18;11(4):358. doi: 10.3390/v11040358 (PMC6520742; doi:10.3390/v11040358)
Supplement: Supplementary file 1 [file viruses-11-00358-s001.zip › viruses-475811-suppl-final/Supplementary Figures 1-6 and Tables 1-4.pdf]

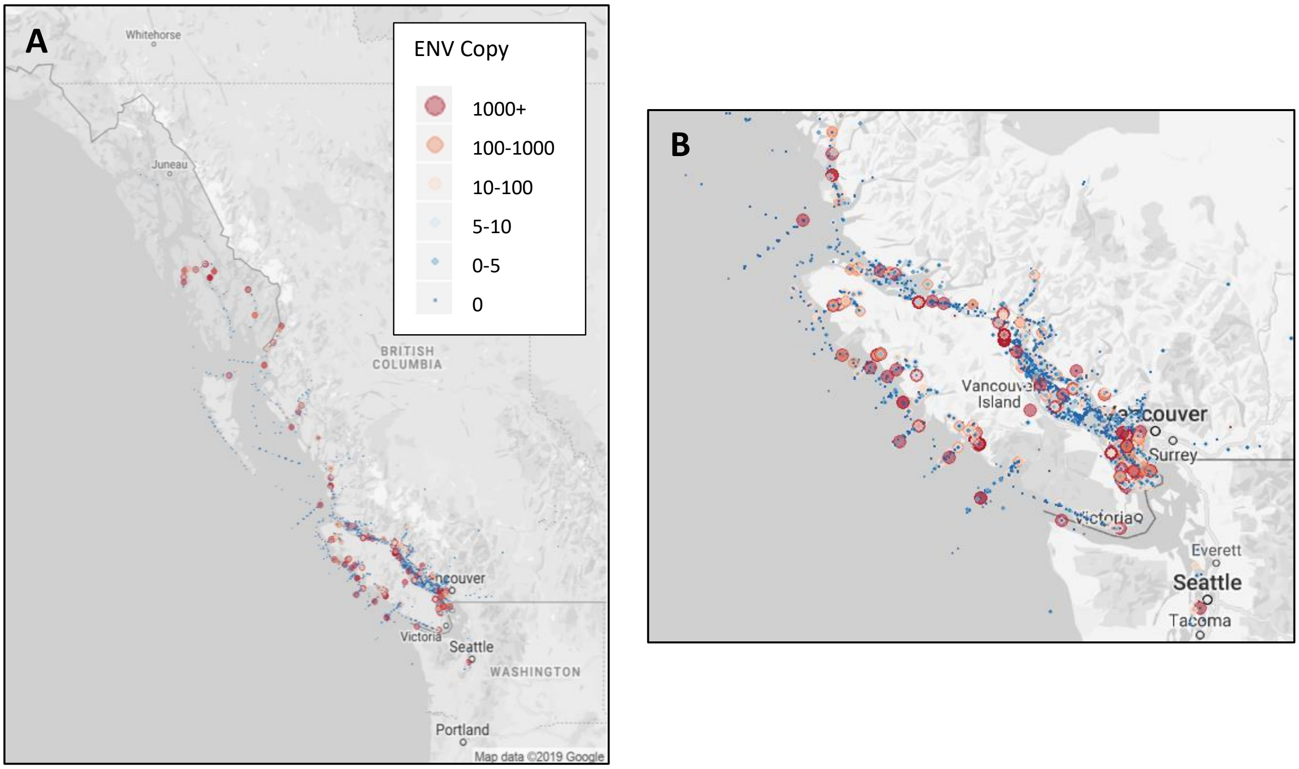

Figure S1: Sampling effort and ENV detection map

(A) Sampling locations, with large dots representing fish with higher ENV copy number. Transparency indicates the number of points at each sampling locations, with darker points representing greater sampling density at a location. **(B)** shows Vancouver Island inset maps. Adults and smolts of all species are shown and LOD criteria are not applied.

Table S1: List of taxa abbreviations used in tables and figures

Aedes taen.	Aedes taeniorhynchus iridescent virus
ATV	Ambystoma tigrinum virus
AVIV	Armadillium vulgare iridescent virus
BM	Brazilian marseillevirus
CQIV	Cherax quadricarinatus iridovirus
ECV	European catfish virus
EHNV	Epizootic haematopoitic necrosis virus
ENV	Erythrocytic necrosis virus
FV3	Frog virus 3
GIV	Grouper Iridovirus
IIV	Invertebrate iridescent virus (various strains)
ISKNV	Infectious spleen and kidney necrosis virus
LCDV	Lymphocystis disease virus (various strains)
LMBV	Largemouth bass/ Santee Cooper ranavirus
LMRV	Lacerta monticola ranavirus
RBIV	Rock Bream iridovirus
RM	Ranavirus maximus
RMIV	Regular mosquito iridescent virus
RSIV	Red Sea Bream iridovirus
SGIV	Singapore Grouper iridovirus
SHIV	Shrimp hemocyte iridescent virus
TRBIV	Turbot reddish body iridovirus

Table S2: ENV BLAST summary

Putative ENV sequences obtained from assembled transcripts of 150 AA or longer which consistently mapped to proteins of similar function when searched by BLAST A) as nucleotide sequence to the NR database B) as predicted proteins from GeneMark to the NR database, C) to the Lymphocystis disease virus transcriptome. A list of taxon abbreviations used is given in Table S1. Columns highlighted in green indicate sequences with BLAST hits to genes which are conserved in all Nucleo-Cytoplasmic Large DNA Virus genomes (Yutin et al. 2009).

SEQ ID	Length (AA)	A) NR BLAST			B) Predicted Protein BLAST			C) LCDV BLAST	
		Taxon	Protein	% Id.	Taxon	Protein	% Id.	Protein	% Id.
82	796	LCDV	Hyp. gp092	30	LCDV	Hyp. gp092	30	Hyp. gp092	30
87	551	LCDV	helicase	38	CQIV	helicase	38	Hyp. helicase	38
89	417	IIV 25	Transcription elongation factor	51	RM	Transcription elongation factor	37	Transcription factor	36
90	431	IIV 22	Hyp. 079L	26	IIV 3	Hyp. 069L	24	N/A	
92	395	SGIV	helicase	50	ATV	helicase	27	N/A	
94	339	CQIV	154R	42	CQIV	154R	47	Replicaton factor or DNA binding/packing protein	43
95	329	GIV	ORF019R	34	CQIV	ORF019R	34	Myristylated membrane protein	31
96	329	IIV 22	Uvr/REP helicase	56	AVIV	Uvr/REP helicase	56	Hyp. 090R	38
98	295	SGIV	RNase III	36	LCDV	RNase III	40	RNase III	41
100	231	LCDV	Hyp. gp057	33	LCDV	Hyp. gp057	32	Hyp. gp057	32
101	228	GIV	004L	38	CQIV	004L	28	SWI/SNF2 helicase	32
102	218	TRBIV	Cytosine DNA methyl-transferase	57	ISKNV	Cytosine DNA methyl-transferase	58	DNA methyltransferase	48
103	212	LCDV	Deoxynucleoside kinase	31	CQIV	Deoxynucleoside kinase	35	Deoxynucleoside kinase	31
104	205	CQIV	103L	49	CQIV	103L	49	Papain-like proteinase	46
105	189	CQIV	141L	42	CQIV	141L	42	N/A	
107	176	LCDV	Proliferating cell nuclear antigen	30	LCDV	Proliferating cell nuclear antigen	30	Proliferating cell nuclear antigen	30
**Contigs with similar functionality**									
DNA repair exonuclease									
83	712	CQIV	DNA repair exonuclease	46	BM	Put. DNA repair exonuclease	51	Hyp. gp105	32
84	662	CQIV	116L	27	CQIV	116L	29	N/A	
85	662	CQIV	116L	27	IIV6	116L	29	N/A	
91	397	GIV	125R	32	IIV6	Flap endonuclease	30	putative XPG/RAD2-type nuclease	28
22	166	CQIV	Repair exonuclease subunit SbcCD	54	IIV6	244L	40	N/A	
DNA dependent RNA polymerase									
77	1491	CQIV	DNA dep. RNA polymerase (alpha)	38	LCDV	DNA directed RNA polymerase	38	RNA dependent RNA polymerase	38
78	1360	LCDV	DNA dep. RNA polymerase	38	ENV	DNA-dependent RNA polymerase,	100	RNA polymerase beta subunit	38
early iridovirus protein									
81	798	CQIV	Hyp. 079L	29	CQIV	Hyp. 079L	29	Hyp. 128R	29
106	181	LCDV	EIP	32	LCDV	EIP	33	EIP	33
21	176	CQIV	Hyp. 079L	32	SHIV	ICP-46	32	Hyp. 128R	27
Phosphotransferase									
97	311	CQIV	144L	26	CQIV	144L	25	phosphotransferase	29
99	278	SGIV	Phospho-transferase	35	LCDV	Phospho-transferase	29	phosphotransferase	32
93	362	CQIV	144L	26	CQIV	144L	25	Phosphotransferase	29
RNA dependent DNA polymerase activity									
11	336	LCDV	RNA dep DNA pol	47		N/A		N/A	
16	265	LCDV	RNA dep DNA pol	44	LCDV	RNA dep DNA pol	44	RNA dep DNA pol	
17	245	LCDV	RNA dep DNA pol	44		N/A		N/A	

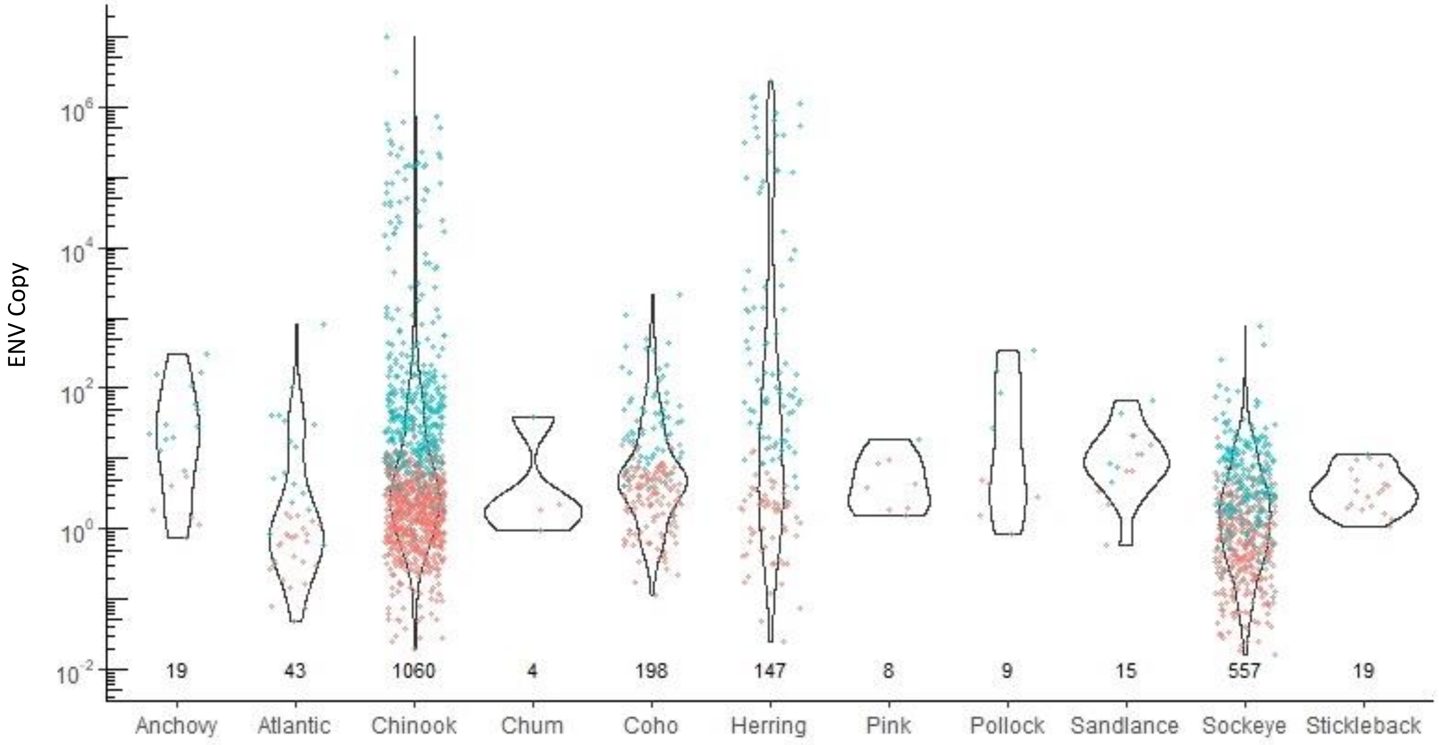

Figure S2: ENV copy number in mixed tissue samples by species.

Blue points indicate values with LOD criteria applied and coral points indicate copy number values which did not pass LOD criteria. Values indicate the total number ENV detections (no LOD criteria).

Table S3: Summary of p values from pairwise tests for differences in load (L) and prevalence (P) among species
Species with higher average loads and prevalence of ENV are listed as columns (top) and species with lower load and prevalence are shown as rows (left). Only smolt with LOD criteria applied were included in analysis.

**Species**	Coho		Herring		Chinook		Sockeye	
	**L**	**P**	**L**	**P**	**L**	**P**	**L**	**P**
Coho			7.4e-05	2.2e-58		1.3e-12		1.7e-05
Chinook			1.7e-05	1.8e-41				
Pink				1.1e-28		3.9e-05		7.1e-04
Sockeye	1.7e-12		3.2e-40	7.8e-48	2.6e-42	2.0e-02		
Atlantic	3.9e-02	9.6e-08	2.1e-06	1.2e-80	3.6e-03	1.1e-35		2.9e-23
Chum				7.1e-25		3.8e-04		2.9e-03

Table S4: Summary of p values and sample size for tests reported in this study

Sample sizes are listed for pairwise comparisons (A) and for species comparisons (B). Only smolt with LOD criteria were used in analysis except for tests comparing age class differences and monthly prevalence (in which both adults and smolt were examined with LOD criteria applied). In general linear models, adults are not removed from analysis explicitly, but age class is considered as a random effect to account for variation among smolt and adult fish.

A) Statistical Tests				
**Variable**	**Category**	**Test**	**P value**	**Finding**
ENV Load	Species	Kruskal-Wallis	6.1e-14	Post-hoc Dunn test
ENV Prevalence	Species	Chi-Square	<2.2e-16	Post-hoc Fisher Exact test
ENV Load	Age class	Kruskal-Wallis	1.8e-04	Adult (n=164) > Smolt (n=837)
ENV Prevalence	Age Class	Chi-Square	1.2e-17	Adult (164/1730 detections) > Smolt (812/17312 detections)
ENV Prevalence	Age Class	General Linear Mixed Effects	1.93e-06	Adult > Smolt *<br>*This effect only occurs in salmon species
ENV Prevalence	Age Class	Chi-Square	1.5e-11	Salmon Species: Adult (120/1485 detections) > Smolt (724/17074 detections)
ENV Prevalence	Age Class	Chi-Square	4.5e-06	Herring: Smolt (88/238 detections) >Adult (44/245 detections)
ENV Prevalence	Habitat	Chi-Square	< 2.2e-16	SW (811/13790 detections) > FW (1/3622 detections)
ENV Prevalence	Habitat	General Linear Mixed Effects	5.5e-08	SW > FW
ENV Prevalence	Monthly	Spearman Correlation	0.004	Sockeye (n=4817) α Atlantic (n=2450)
ENV Prevalence	Yearly	Chi-Square	<2.2e-16	Post-hoc Chi Squared tests with Bonferroni correction

B) Sample Size by Species (with LOD)		
**Species**	**Adults**	**Smolts**
Atlantic	1310	2454
Chinook	259	6848
Chum	0	191
Coho	13	3652
Herring	249	289
Pink	0	233
Sockeye	4	4869

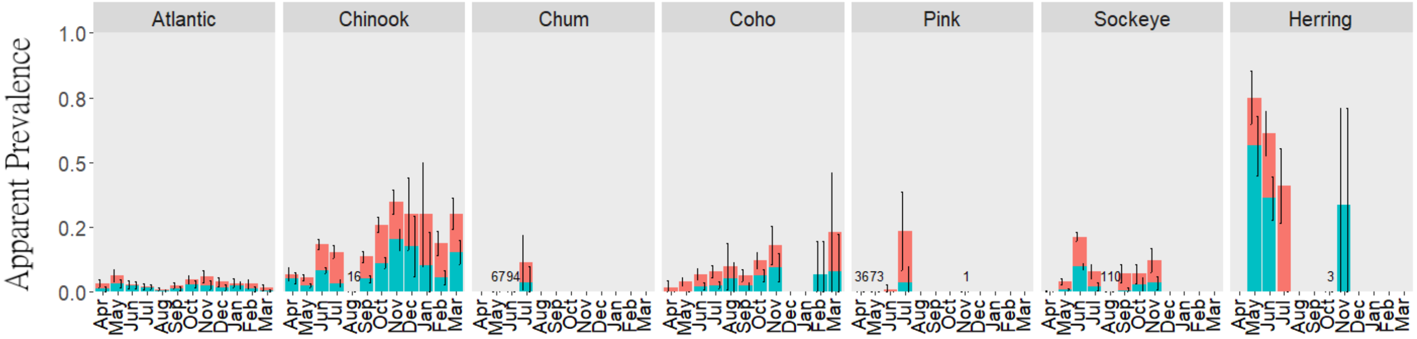

Figure S3: Seasonal ENV prevalence by species

Samples with LOD criteria applied are in blue and samples without LOD criteria applied are in coral, with relevant species indicated at the top. Printed values indicate sample sizes for months when prevalence was zero. Error bars indicate 95% confidence intervals. For aquaculture fish, both smolts and adults are shown. Only smolts are shown for wild fish.

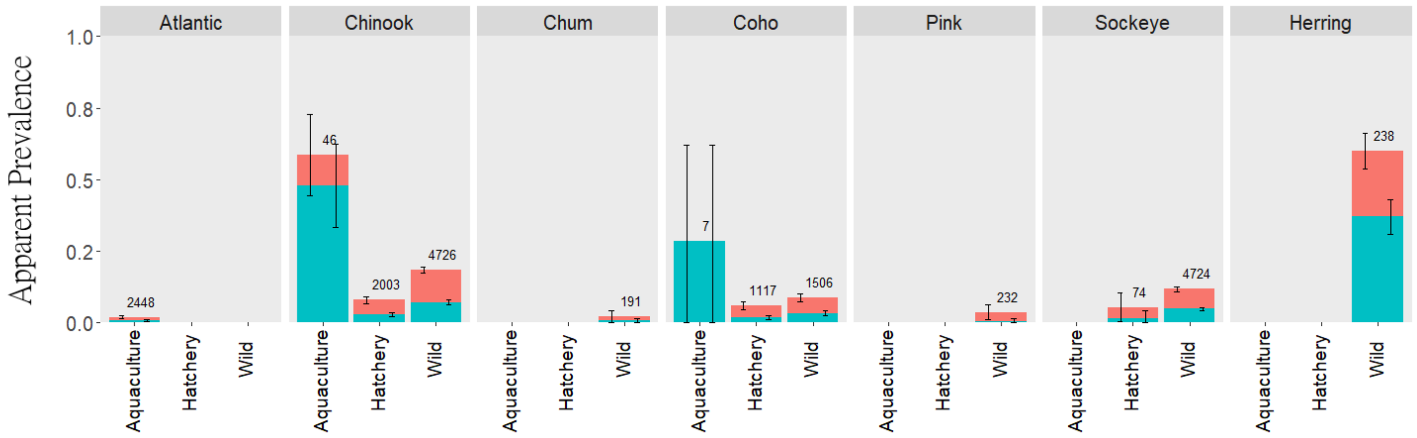

Figure S4: ENV prevalence by population

Samples with LOD criteria applied are in blue and samples without LOD criteria applied are in coral, with relevant species indicated at the top. Printed values indicate sample sizes and error bars indicate 95% confidence intervals. Hatchery fish represent fin-clipped hatchery fish and only smolts are shown.

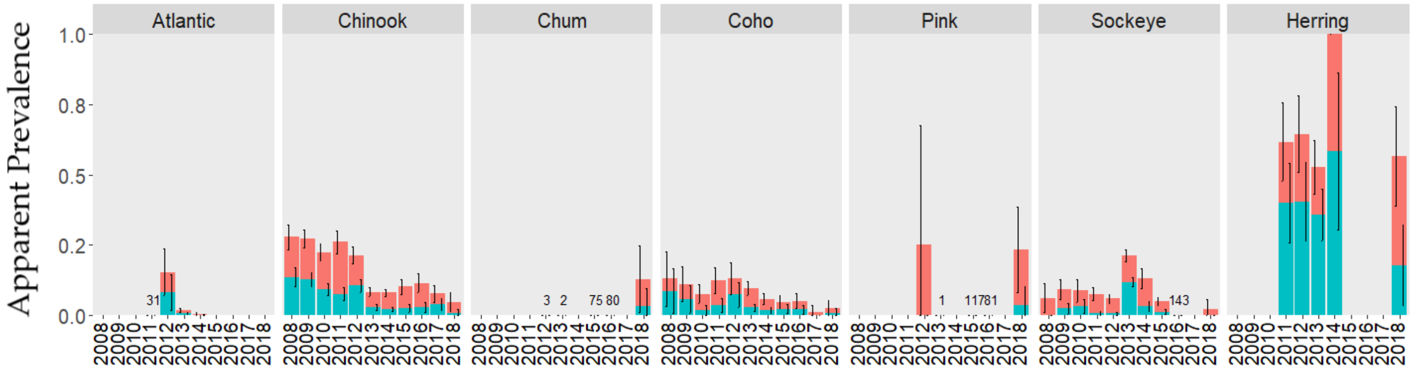

Figure S5: ENV prevalence by year and species

Samples with LOD criteria applied are in blue and samples without LOD criteria applied are in coral, with relevant species indicated at the top. Printed values indicate sample sizes for years with a prevalence of zero and error bars indicate 95% confidence intervals. Only smolts are shown.

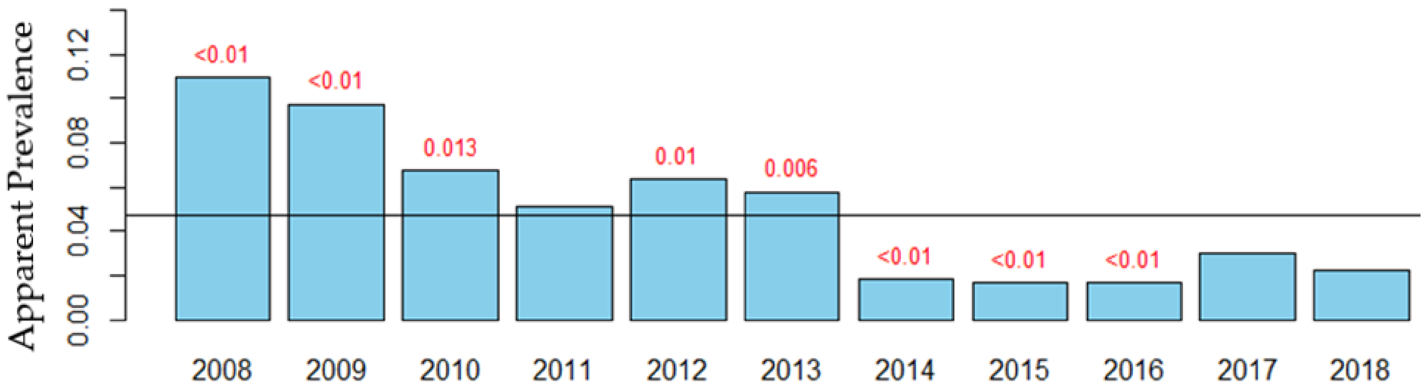

Figure S6: ENV prevalence by year

Total ENV prevalence by year for all salmon species and herring, showing only smolts with LOD criteria applied. Red values indicate significant p values from multiple Chi-squared Goodness of fit tests against the average prevalence (black line) with Bonferroni corrections applied. In years for which p values are not listed, ENV prevalence did not differ significantly from the overall average prevalence.
